# *Ex vivo* modeling of nasal epithelial airway inflammation in respiratory viral infections

**DOI:** 10.3389/fmicb.2026.1819581

**Published:** 2026-06-03

**Authors:** Leila Fotoohabadi, Arnaud John Kombe Kombe, Pratik Lama Tamang, Steven Klawinsky, Matthew Mitakidis, Kamilla Ablyazova, Richard Pyles, Theodoros Kelesidis

**Affiliations:** 1 Division of Infectious Diseases and Geographical Medicine, Department of Medicine, University of Texas Southwestern Medical Center, Dallas, TX, United States; 2 Department of Microbiology and Immunology, University of Texas Medical Branch School of Medicine, Galveston, TX, United States; 3 Department of Immunology, University of Texas Southwestern Medical Center, Dallas, TX, United States

**Keywords:** host airway cell responses, human rhinovirus infection, influenza, nasal airway cell culture model, nasal airway inflammation, respiratory viral infections

## Abstract

**Background:**

Early inflammatory responses at the nasal airway epithelium, the primary portal of entry for respiratory viruses, remain unclear. Primary airway cell culture models have yielded contradictory results due to heterogeneity among donors and variable processing of airway cells.

**Methods:**

Using a novel polarized *ex vivo* culture system of primary, immortalized human nasal epithelial cells (hNECs) in air-liquid interface (ALI) culture, we compared inflammatory responses following infection with human rhinovirus (HRV16) or influenza virus.

**Results:**

Over 24 and 48 h, both HRV16 and H1N1-PR8 consistently and similarly increased levels of apical and basal TNF-α, and VEGF. HRV16 differentially increased secretion of IL-1β, IL-6, IL-8, and IL-10 levels in apical or basal surfaces compared to H1N1-PR8. Both HRV16 and H1N1-PR8 induced significant upregulation of VEGF, a mediator of angiogenesis. Both HRV16 and H1N1-PR8 induced much greater upregulation of apical rather than basal IL-6 secretion.

**Conclusion:**

Our data provide mechanistic insight into the pro-inflammatory effects of respiratory viruses on nasal secretions and show that both HRV and influenza A induce a marked increase in the secretion of IL-1β, IL-6, and VEGF from human nasal airway epithelium, offering a possible mechanism for influenza-mediated nasal congestion. This model enables controlled investigation of early nasal epithelial host–virus interactions in physiologically relevant polarization, to explore host-virus nasal airway interactions.

## 1 Introduction

Respiratory viral infections are a significant cause of morbidity and mortality worldwide. Respiratory viruses such as human rhinovirus (HRV) and influenza A enter the human airways through droplets and invade the nasal airway epithelium, where they replicate and induce associated cell and tissue damage by releasing proinflammatory cytokines and chemokines such as Interleukin-6 (IL-6), Tumor necrosis factor alpha (TNF-α), and vascular endothelial growth factor (VEGF). The nasal mucosa provides the first line of defense against inhaled pathogens. The nose is an attractive source of airway epithelial cells, particularly in populations where bronchoscopy may not be possible, because nasal airway cells exhibit inflammatory responses to the host airways similar to those of bronchial airway cells (McDougall et al., 2008). Therefore, nasal epithelial cultures constitute an accessible surrogate for studying lower airway inflammation. Thus, understanding the immunopathogenesis of respiratory viral infections at the level of the nasal airway is critical to developing novel therapeutics with antiviral and anti-inflammatory effects.

However, little is known about the differential impact of respiratory viruses on polarized proinflammatory responses of apical airway nasal epithelial cells, which differ from those of basal airway nasal epithelial cells that contribute to the mucosal barrier. There are several bottlenecks in studying respiratory viruses in human nasal airways, including limited access to human nasal airway tissues, the inability to study different respiratory viruses sequentially in the same person because they infect at different times, and significant biological heterogeneity among individuals. The structure of the human nasal airway epithelium differs markedly from that of animal nasal airways, and certain respiratory viruses, such as H1N1 influenza and HRV, cannot infect animal nasal airway epithelium. This is why the literature is limited to the differential impact of respiratory viruses on proinflammatory human nasal airway responses.

Prior studies on host airway responses of nasal airway cells in the setting of respiratory viruses used primary cell culture systems in which patient-derived nasal epithelial cells are differentiated at an air–liquid interface (ALI) to model infection of the nasal epithelium, where respiratory viruses initially replicate (Brocke et al., 2025; Gard et al., 2021; Otter et al., 2024; Tan et al., 2018; Vanderheiden et al., 2020). After differentiation, nasal ALI cultures recapitulate various features of the *in vivo* nasal epithelium, including its heterogeneous cellular population and mucociliary clearance function. However, primary cells must be freshly isolated from donors and possess a finite proliferative capacity, entering replicative senescence after only a few population doublings and losing key differentiation features with extended culture, making them difficult to expand, impossible to freeze and reuse, and inherently variable between donors and passages, which complicates coordination and reproducibility across experiments and laboratories (Levardon et al., 2018). This has led to significant heterogeneity in the literature across studies regarding mechanistic data on the specific effects of different respiratory viruses on nasal airway cells, such as apical versus basal secretion of proinflammatory cytokines. For example, in primary differentiated nasal epithelial cultures infected with SARS-CoV-2 or other respiratory viruses, the magnitude and kinetics of interferon responses vary widely between reports (Hatton et al., 2021). Prior studies of primary nasal, nasopharyngeal, and bronchial epithelial ALI cultures found that there are also donor-specific cytokine responses to the same stimuli, showing heterogeneity even within the same experimental setup (van Woudenbergh et al., 2025). Studies comparing pediatric and adult primary nasal epithelial cells have shown conflicting host airway responses and age-dependent variability in host airway cell signaling (Zhu et al., 2022). Other studies with airway primary cells further highlighted that primary cells don’t always produce uniform immune responses even in response to identical stimuli (van Woudenbergh et al., 2025). Because primary epithelial cultures are derived from different donors with variable genetic backgrounds, anatomical sites, ages, and environmental exposures, and are differentiated under non-identical conditions, the same pathway (e.g., inflammatory cytokine signaling) can appear up-regulated in one study but not another. This leads to contradictory interpretations in the literature and underscores challenges in using primary nasal airway cells as a standardized model system.

In contrast, immortalized airway basal cell lines engineered, for example, via telomerase expression, retain the ability to proliferate over dozens of passages while maintaining multipotent differentiation into ciliated, secretory and goblet cell types under ALI conditions, enabling consistent generation of polarized epithelia without repeated primary harvests (Walters et al., 2013). Because immortalized cells can be expanded long-term, cryopreserved, and provide homogeneous populations with reduced donor variability, they facilitate more standardized, scalable studies and reliable gene editing or pharmacologic testing, reducing technical difficulty and cost relative to the laborious isolation and culture coordination required for primary nasal ALI models (Levardon et al., 2018).

To address these gaps in knowledge and to conduct controlled studies of the human nasal ecosystem in the setting of independent respiratory viral infections, we have utilized a previously developed novel *ex vivo* mucosal model created by immortalized human nasal epithelial cells (hNEC) established from three human donors that were cultured with an air-liquid-interfaced (ALI), apical surface on a porous transwell membrane (Charles et al., 2019). The immortalized nature of the NEC from several haplotypes of both genders eliminates the limitations associated with primary cell senescence and allows for repeated culture studies, avoiding major limitations of other cell culture models, such as limited availability of human donors and major biological heterogeneity among different donors. We utilized this mechanistic airway model to determine the direct impact of HRV and H1N1 influenza in nasal airway tissue levels of key mediators of tissue injury (inflammation) during nasal airway infection. Thus, to understand the crosstalk between nasal airway inflammation and respiratory viral infection, we measured the protein levels of secreted IL-1β, IL-6, IL-8, IL-10, TNF-α, and VEGF at the apical and basal surfaces of the hNEC ALI airway culture system.

## 2 Materials and methods

### 2.1 Materials

The following materials were purchased and used throughout the study; Costar 96-Well, Cell Culture-Treated, Flat-Bottom Microplate (Corning, Cat#07-200-91), Greiner Bio-One, Cell Culture Microplate, 96 Well, Ps, Flat-bottom, Clear, Black (Cat#655090), Dulbecco’s modified eagle medium (DMEM) (Gibco, Cat# 10-569-044), Dulbecco’s Modified Eagle Medium/Nutrient Mixture F-12 (DMEM/F-12) (Gibco, Cat#12634010), Airway Epithelial Cell Basal Medium (ATCC, Cat# PCS-300-030), Bronchial Epithelial Cell Growth Kit, (ATCC, Cat# PCS-300-040), and Collagen type I (Corning Cat# 354249), Bio Rad (BCA) protein assay kit (Cat# PI23227), Penicillin/Streptomycin 100X (Cat# 15-140-122), sterile nylon 40 μm Filter (Cat# 07-201-430), T-PER™ tissue protein extraction reagent (Cat# PI78510), and trypsin-ethylenediamine tetraacetic acid (EDTA) (0.25% w/v), Human Nasal Epithelial Cells (HNEpC) PromoCell (Cat.No. C-12620).

### 2.2 Cells

H1 Hela epithelial cells (Cat# CRL-1958) and Madin-Darby Canine Kidney (MDCK) cells (Cat# CCL-34) were purchased from American Type Culture Collection (ATCC (Manassas, VA) and were utilized to propagate the HRV16 and H1N1-PR8 viruses, respectively. All cells were maintained at 37°C and 5% CO2 in DMEM or MEM supplemented with 10% (v/v) FBS, penicillin (100 units/mL), and streptomycin (100 μg/mL) (1X P/S).

### 2.3 Viruses

Human rhinovirus 16 strain 11757 (Cat# VR-283), and Influenza A virus (H1N1) strain A/PR/8/34 (Cat# VR-95) were procured from ATCC.

### 2.4 hNEC ALI cell culture model

hNEC cells from three human donors were immortalized with HPV E6/E7 as previously described and were kindly provided by Dr. Pyles (Charles et al., 2019). All immortalized cells were propagated in ATCC airway medium supplemented with 5% CO2 at 37°C. Cultures were refed every other day. Passaging was performed every 2–3 days upon reaching 80% confluence. Cells were transferred to collagen-I-coated Transwell inserts (Corning, Cat#3470) and maintained at a liquid-liquid interface (LLI) in supplemented ATCC airway medium for 2–3 days until confluence was achieved. Differentiation of hNEC under ALI culture conditions was carried out in 1:1 supplemented ATCC airway medium and DMEM/F12 for 28 days. The medium in the basal chamber of all cultures was replaced every other day. Differentiation of hNEC ALI cells was checked on day 28 using Real-Time quantitative Polymerase Chain Reaction (RT-qPCR) for the differentiation genes FOX-J1, centrosomal protein 110 (Cp110), and cadherin-related family member 3 (CDHR3) that are not expressed in basal undifferentiated cells and are important for viral infections (Everman et al., 2019; Lai et al., 2011) as well as for the MUC5AC gene (Mucin 5AC, Oligomeric Mucus/Gel-Forming) which encodes a major gel-forming mucin protein primarily secreted by surface goblet cells in the airway epithelium to protect against pathogens and is a marker for goblet cell differentiation in nasal and airway cells (Wang et al., 2012).

These genes predict airway epithelial cell differentiation and function and are established mediators and surrogate measures of ciliation and differentiation in independent experimental disease models (Bochkov et al., 2015; Guo et al., 2026; Mertens et al., 2017; Tsang and Dynlacht, 2013; You et al., 2004).

### 2.5 Respiratory viral infection in ALI cultures

All studies involving live viruses were conducted at the high-containment facility with appropriate institutional biosafety approvals. HRV16 and H1N1 were passaged in H1 HeLa and MDCK cells, respectively, and viral stocks were aliquoted and stored at –80°C until use. Viral titer was measured in H1HeLa (HRV16) and MDCK (H1N1-PR8) cells using the median tissue culture infectious dose (TCID_50_) assay. Infectious titers were quantified by limiting dilution titration using H1HeLa and MDCK cells. Briefly, target cells were seeded at 10,000 cells/well in 96-well plates. The next day, the virus-containing supernatant was applied at serial 10-fold dilutions from 10^–1^ to 10^–8^, and after 3–5 days, viral cytopathic effect (CPE) was assessed by microscopy or by measuring cytopathic damage using the lactate dehydrogenase (LDH) assay in cell culture supernatants. TCID_50_/mL was calculated using the Reed-Muench method. Fully differentiated nasal hNEC ALI cultures were infected with HRV16 and/or H1N1-PR8 at a multiplicity of infection (MOI) of 0.01. To determine whether differential host airway inflammatory responses are secondary to differential effects on host airway cells rather than to differences in viral inoculum, we used an identical MOI (0.01).

### 2.6 hNEC *in vitro* cell culture model and respiratory viral infections

Commercially available hNEC cells and immortalized hNEC were propagated in ATCC airway medium supplemented with 5% CO2 at 37°C, then seeded into 96-well culture plates. Cells were infected upon reaching at least 70% confluence with HRV16 and H1N1 at MOIs of 0.01 and 0.1. Intracellular viral RNA levels were quantified by qRT-PCR using the comparative ΔΔCt method as an established surrogate measure of relative viral replication/infectivity under standardized infection conditions.

### 2.7 Immunoassays

Protein content per each ALI insert was determined based on the Bicinchoninic acid assay (BCA) assay as previously described (Daskou et al., 2022). Human IL-1β, IL-6, IL-8, IL-10, TNF-α, and VEGF were determined in apical and basal cell culture supernatants using the human magnetic Luminex performance assay kits according to the manufacturer’s instructions (R&D) and as previously described (Daskou et al., 2022). The limit of detection of the Luminex immunoassay for the least abundant measured protein (IL-10) was 2 pg/mL.

### 2.8 RNA extraction and real-time quantitative reverse transcription polymerase chain reaction (RT-qPCR)

Total RNA was isolated using the RNeasy Mini Kit or Direct-zol RNA Miniprep kit (Zymo Research), and complementary deoxyribonucleic acid (cDNA) was synthesized. RT-qPCR was performed using SYBR Green Master Mix and primers listed in Table 1. All qRT-PCR reactions were performed using BIO-RAD CFX96 Real-Time PCR Detection System (Bio-Rad Laboratories, Hercules, CA) on 96-well plates. PCR reactions included SYBR Green RT-PCR Master Mix, 5 μM primers, and 5 μL of cDNA. Reactions were incubated at 45°C for 10 min for reverse transcription, 95°C for 2 min, followed by 40 cycles of 95°C for 15 s (sec) and 60°C for 60 s. Gene expression fold change was calculated with the Delta-delta-cycle threshold (DDCt) method. RNA levels were normalized to GAPDH as an endogenous control and expressed as fold change relative to uninfected samples.

**TABLE 1:** List of primers.

Target	Forward primer (5′–3′)	Reverse primer (5′–3′)
IL-1β	GCAGCAGCACATCAACAAGAG	CCACGGGAAAGACACAGGTAG
il-8	GAGAGTGATTGAGAGTGGACCAC	CACAACCCTCTGCACCCAGTTT
IL-6	GGTACATCCTCGACGGCATCT	GTGCCTCTTTGCTGCTTTCAC
TNF-α	CCCAGGCAGTCAGATCATCTTC	AGCTGCCCCTCAGCTTGA
VEGF	TTGCCTTGCTGCTCTACCTCCA	GATGGCAGTAGCTGCGCTGATA
FOX-J1	ACTCGTATGCCACGCTCATCTG	GAGACAGGTTGTGGCGGATTGA
CCP110	TGTCTCAAGCGGACTCACTCCA	CCAGAGGTAGAATGGTGCTTCG
GAPDH	CCACCTTTGACGCTGGG	CATACCAGGAAATGAGCTTGACA
MUC5AC	CCGGAGGTGAGCATCGAACA	TCACAGGTGTGGAGGTCACG

### 2.9 Statistics

Unless noted, error bars in all figures represent mean and standard error of means (SEM). In the figures, *p*-values for comparisons between groups are presented and denoted by asterisks. Each experiment includes at least 3 biological replicates (i.e., wells) per human donor, and data points from 3 independent human donors are shown. The Mann-Whitney U test was used to assess statistical differences between 2 groups. *P* < 0.05 were considered significant. Our study, largely exploratory, was not powered to detect effect sizes with adjustment for multiple comparisons. Rather, consistency, direction, and magnitude of the effect in conjunction with the nominal *P*-values were considered to help distinguish true− and false−positive findings (Feise, 2002). All analyses were performed using GraphPad version 10.0 (GraphPad Software, San Diego, CA).

## 3 Results

### 3.1 hNEC differentiation

**FIGURE 1:**
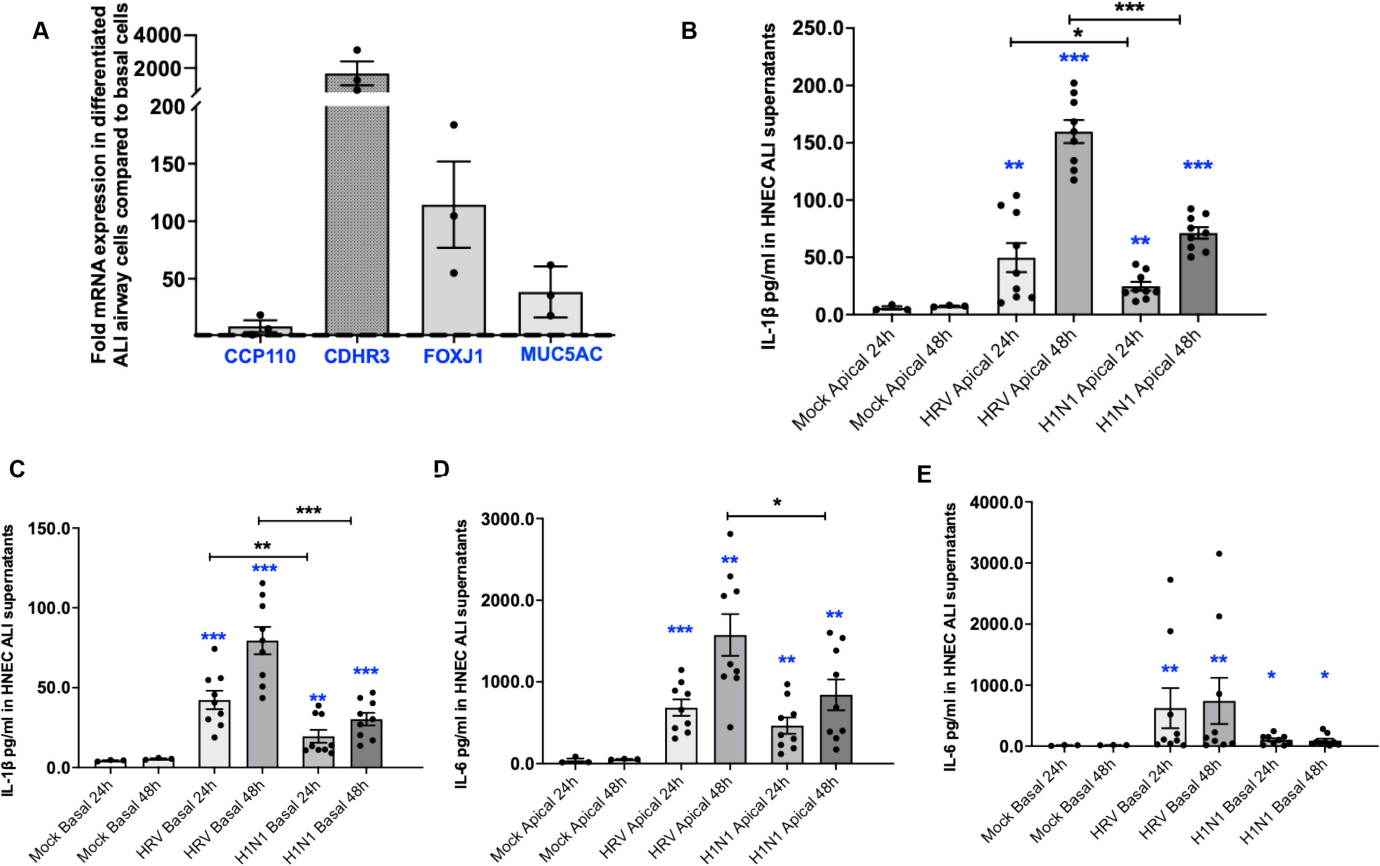
Differentiation of nasal airway cell cultures and impact of HRV and influenza infection on mediators of airway inflammation (IL-1β and IL-6) in an *ex vivo* model of nasal airways. Human nasal epithelial cells (HNECs) from three human donors were prepared and infected with HRV and H1N1 for 24 and 48 h, as described in the methods. **(A)** hNEC differentiation was confirmed by qPCR using four independent differentiation genes that are not expressed in non-ciliated basal undifferentiated cells (FOX-J1, CCP-110, CDHR3) and for MUC5AC as described in Methods. **(B–E)** Proinflammatory cytokines interleukin 1 beta (IL-1β) **(B,C) and** IL-6 **(D,E)** were determined by multiplex Luminex immunoassay in both apical **(B,D)** and basal **(C,E)** chambers of air-liquid interface (ALI) inserts as described in methods. All panels show summary data as mean ± standard error of the mean (SEM) from at least 3 biological replicates per donor. Three HNEC ALI cultures were prepared per human donor, and data from 3 donors are shown. Each data point represents the average of at least two technical replicates per one biological sample. Statistical comparisons between the uninfected control group and each experimental group were performed using a two-tailed Mann–Whitney test and are indicated by a blue asterisk. A statistical comparison between two non-control experimental groups was performed using a two-tailed Mann–Whitney test, and the result is indicated by a black asterisk. The *p*-value for each comparison is shown above each column (*p < 0.05, **p < 0.01, ***p < 0.001). Alt Text: Graph and data showing measures of nasal airway differentiation and inflammation in human nasal airway cell culture models infected with influenza or human rhinovirus over 48 h compared to uninfected cell cultures.

hNEC differentiation was confirmed by qPCR using three independent differentiation genes that are not expressed in non-ciliated basal undifferentiated cells (FOX-J1, CCP-110, CDHR3) and the MUC5AC gene, a marker for goblet cell differentiation. There was a mean 8.8-fold upregulation in mRNA levels of CCP-110, a mean 1,673-fold upregulation in mRNA levels of CDHR3, a mean 114-fold upregulation in mRNA levels of FOX-J1, and a mean 39-fold upregulation in mRNA levels of MUC5AC compared to non-expression of these genes in undifferentiated cells (*p* < 0.01 for all comparisons) (Figure 1A).

### 3.2 Viral infection

In nasal airway epithelial cell cultures, the HRV16 replication peaked at 24 h post-infection (h.p.i) (mean viral titer 2.1* 10^4^ PFU/mL), while for H1N1 the mean viral titer was 1.2*10^^2^ PFU/mL. H1N1 replication peaked at 48 h.p.i (mean viral titer 1.9* 10^^4^ PFU/mL), while for HRV16 the mean viral titer was 1.3*10^^3^ PFU/mL. To determine whether immortalization of hNEC affected their relative infectability to HRV16 and H1N1 compared to primary hNEC, we performed a tenfold infection with increasing viral MOIs (0.01 and 0.1) over 24 h. *In vitro* infection of primary and immortalized hNECs with increasing amounts of viruses showed a similar magnitude of increasing relative infection for both HRV (Supplementary Figure 1A) and H1N1 (Supplementary Figure 1B). These results showed that the relative infectability of HRV16 (as measured by intracellular gene levels and the qPCR DDCT method) at 24 h.p.i was on average 1.41 fold higher in primary hNECs and 1.44 folder higher in immortalized hNECs compared to H1N1, respectively, and also confirmed the viral titer results in immortalized nasal airway epithelial cell cultures at 24 h.p.i, which showed that the relative infectability of HRV16 was on average 1.82 fold higher compared to H1N1.

### 3.3 Inflammatory responses induced by HRV compared to uninfected cells

**FIGURE 2:**
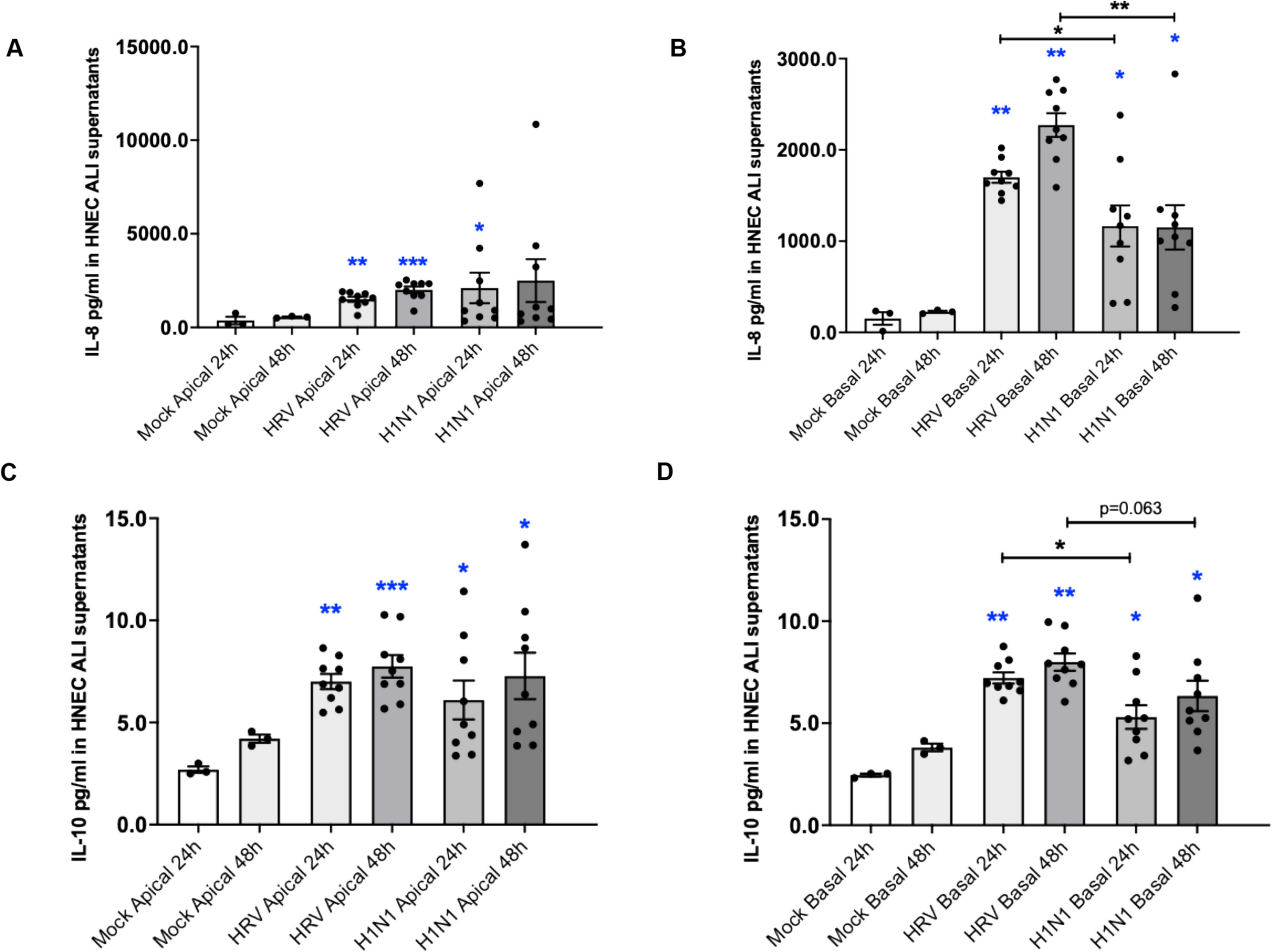
Impact of HRV and influenza infection on mediators of airway inflammation (IL-8, IL-10) in an *ex vivo* model of nasal airways. Human nasal epithelial cells (HNECs) from three human donors were prepared and infected with HRV and H1N1 for 24 and 48 h, as described in the methods. **(A–D)** Proinflammatory cytokines interleukin 8 (IL-8 **(A,B)** and IL-10 **(C,D)** were determined by multiplex Luminex immunoassay in both the apical **(A,C)** and basal **(B,D)** chambers of air-liquid interface (ALI) inserts, as described in the methods. All panels show summary data as the mean ± standard error of the mean (SEM) from at least 3 biological replicates per donor. Three HNEC ALI cultures were prepared per human donor, and data from 3 donors are shown. Each data point represents the average of at least two technical replicates per one biological sample. Statistical comparison between the uninfected control group and each experimental group was performed using a two-tailed Mann–Whitney test and is indicated by a blue asterisk. A statistical comparison between two non-control experimental groups was performed using a two-tailed Mann–Whitney test and is indicated by a black asterisk. The *p*-value for each comparison is shown above each column (*p < 0.05, **p < 0.01, ***p < 0.001). Alt Text: Graph and data showing measures of nasal airway inflammation in human nasal airway cell culture models infected with influenza or human rhinovirus over 48 h compared to uninfected cell cultures.

**FIGURE 3:**
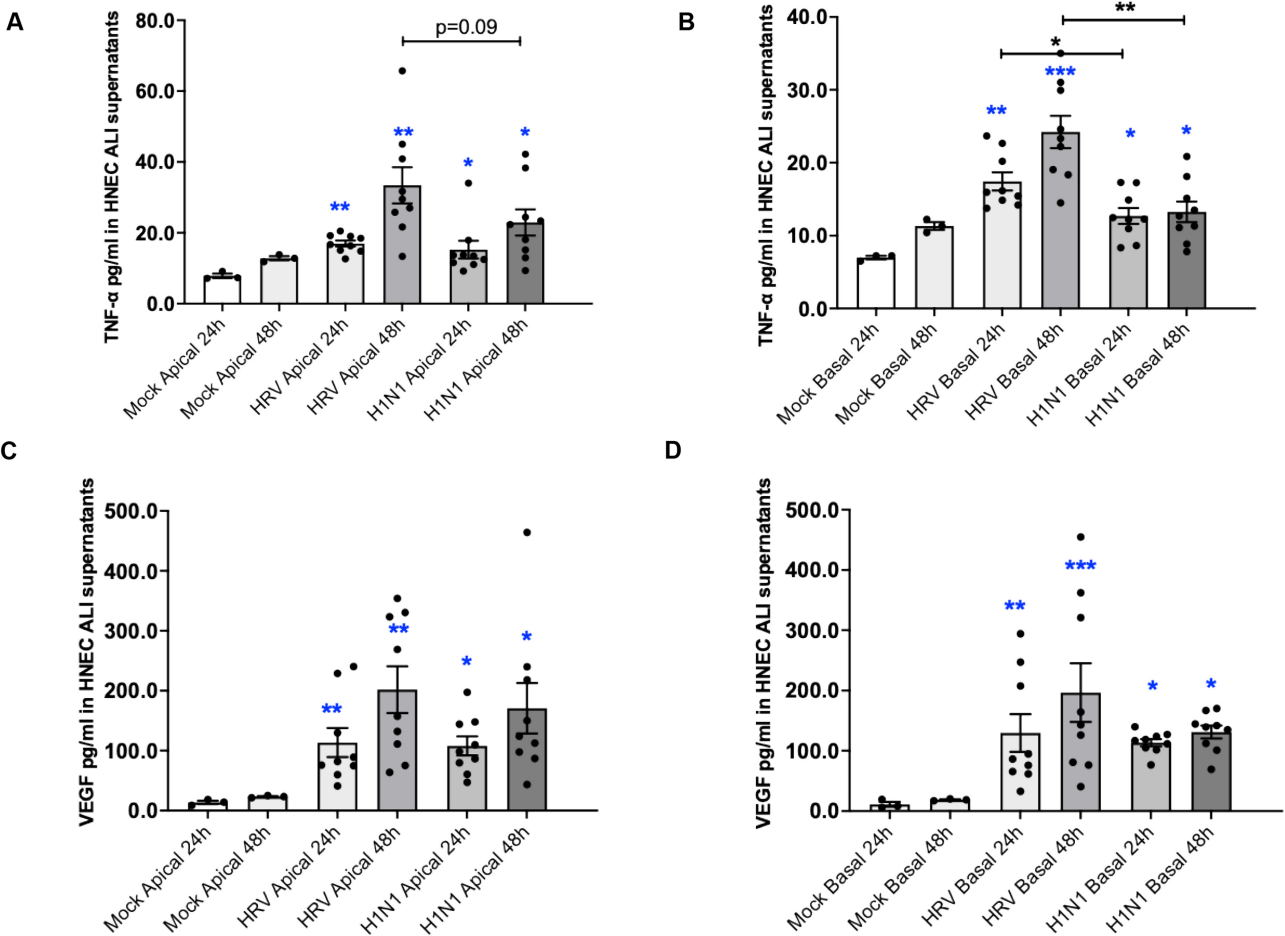
Impact of HRV and influenza infection on mediators of airway inflammation (TNF-α, VEGF) in an *ex vivo* model of nasal airways. Human nasal epithelial cells (HNECs) from three human donors were prepared and infected with HRV and H1N1 for 24 and 48 h, as described in the methods. **(A–D)** Proinflammatory cytokines, tumor necrosis factor alpha (TNF-α) **(A,B)** and Vascular endothelial growth factor (VEGF) **(C,D)**, were determined by multiplex Luminex immunoassay in both the apical **(A,C)** and basal **(B,D)** chambers of air-liquid interface (ALI) inserts, as described in the methods. All panels show summary data as the mean ± standard error of the mean (SEM) from at least 3 biological replicates per donor. Three HNEC ALI cultures were prepared per human donor, and data from 3 donors are shown. Each data point represents the average of at least two technical replicates per one biological sample. Statistical comparison between the uninfected control group and each experimental group was performed using a two-tailed Mann–Whitney test and is indicated by a blue asterisk. A statistical comparison between two non-control experimental groups was performed using a two-tailed Mann–Whitney test and is indicated by a black asterisk. The *p*-value for each comparison is shown above each column (*p < 0.05, **p < 0.01, ***p < 0.001). Alt Text: Graph and data showing measures of nasal airway inflammation in human nasal airway cell culture models infected with influenza or human rhinovirus over 48 h compared to uninfected cell cultures.

Using a sensitive multiplex Luminex immunoassay based on fluorescence, we found that both basal and apical surfaces of uninfected mock HNEC ALI inserts secreted detectable levels of IL-1β, IL-6 (Figures 1B–E), IL-8, IL-10 (Figures 2A–D), TNF-α and VEGF (Figures 3A–D), and the most abundant cytokine was IL-8, followed by IL-6 (Figures 1B–E, 2A–D, 3A–D). Compared to uninfected HNEC ALI cultures, HRV16 infection at 24 and 48 h consistently increased levels of apical and basal IL-1β (Figures 1B,C), IL-6 (Figures 1D,E), IL-8 (Figures 2A,B), IL-10 (Figures 2C,D), TNF-α (Figures 3A,B), and VEGF (Figures 3C,D). To obtain further mechanistic insight into the time-dependent proinflammatory effects of respiratory viruses in HNEC ALI cultures, we also measured cytokine levels in cell culture supernatants over 48 h post-infection (h.p.i). Compared with 24 h.p.i., HRV16 infection at 48 h.p.i. further increased levels of apical IL-1β (Figure 1B), IL-6 (Figure 1D), IL-8 (Figure 2A), TNF-α (Figure 3A), and VEGF (Figure 3C). Compared with 24 h.p.i., HRV16 infection at 48 h.p.i. further increased levels of basal IL-1β (Figure 1D), IL-6 (Figure 1E), IL-8 (Figure 2B), and TNF-α (Figure 3B). HRV16 infection at 48 h.p.i. did not further increase levels of basal IL-6 (Figure 1E), apical and basal IL-10 (Figures 1C,D) and basal VEGF (Figure 3D).

### 3.4 Inflammatory responses induced by influenza compared to uninfected cells

Compared to uninfected HNEC ALI cultures, H1N1 infection at 24 h also consistently and similarly to HRV16 increased levels of apical and basal IL-1β (Figures 1B,C), IL-6 (Figures 1D,E), IL-8 (Figures 2A,B), IL-10 (Figures 2C,D), TNF-α (Figures 3A,B), and VEGF (Figures 3C,D). Compared with 24 h.p.i., H1N1 infection at 48 h.p.i. further increased levels of apical IL-1β (Figure 1B), IL-6 (Figure 1D), and TNF-α (Figure 3A). Compared with 24 h.p.i., HRV16 infection at 48 h.p.i. further increased levels of basal IL-1β (Figure 1D). H1N1 infection at 48 h.p.i. did not further increase levels of basal IL-6 (Figure 1E), IL-8 (Figure 2B), apical and basal IL-10 (Figures 1C,D), TNF-α (Figure 3B), apical VEGF (Figure 3C), and basal VEGF (Figure 3D).

Compared to uninfected cells, the highest induced inflammatory responses by both HRV16 and H1N1 were the induction of IL-6 and IL-8 ( > 500-fold mean upregulation, *p* < 0.05 for all comparisons (Figures 1D–E, 2A,B). Both HRV16 and H1N1 induced a significant upregulation of apical VEGF ( > 100-fold mean increase, *p* < 0.05 for all comparisons) (Figure 3C).

### 3.5 Inflammatory responses induced by HRV compared to influenza

Next, we determined the differential inflammatory responses induced by HRV compared with influenza in hNEC ALI airway cultures infected with a similar viral inoculum (MOI 0.01 for both infections). HRV16 infection of HNEC ALI inserts at 24 h and at 48 h induced higher secretion of IL-1β in both apical (Figure 1B) and basal (Figure 1C) chambers and higher secretion of TNF-α in apical (Figure 3B) compared to H1N1 infection of HNEC ALI inserts. HRV16 infection of HNEC ALI inserts at 48 h also induced higher secretion of IL-6 in apical chambers compared to H1N1 infection of HNEC ALI inserts (Figure 1D). HRV16 infection of HNEC ALI inserts at 24 and 48 h induced higher secretion of IL-8 in basal chambers compared to H1N1 infection of HNEC ALI inserts (Figure 1B). HRV16 infection of HNEC ALI inserts at 24 h induced higher secretion of IL-10 in basal chambers compared to H1N1 infection of HNEC ALI inserts (Figure 2D). HRV16 infection of HNEC ALI inserts at 48 h tended to induce higher secretion of IL-10 (*p* = 0.063) (Figure 2D) in basal chambers and TNF-α in apical chambers (*p* = 0.09 (Figure 3A) compared to H1N1 infection of HNEC ALI inserts. Overall, there was no other significant differential impact of HRV16 versus H1N1 on both apical and basal surfaces in any of the inflammatory mediators (Figures 1B–E, 2, 3).

### 3.6 Polarized inflammatory responses of apical versus basal airway epithelium

**FIGURE 4:**
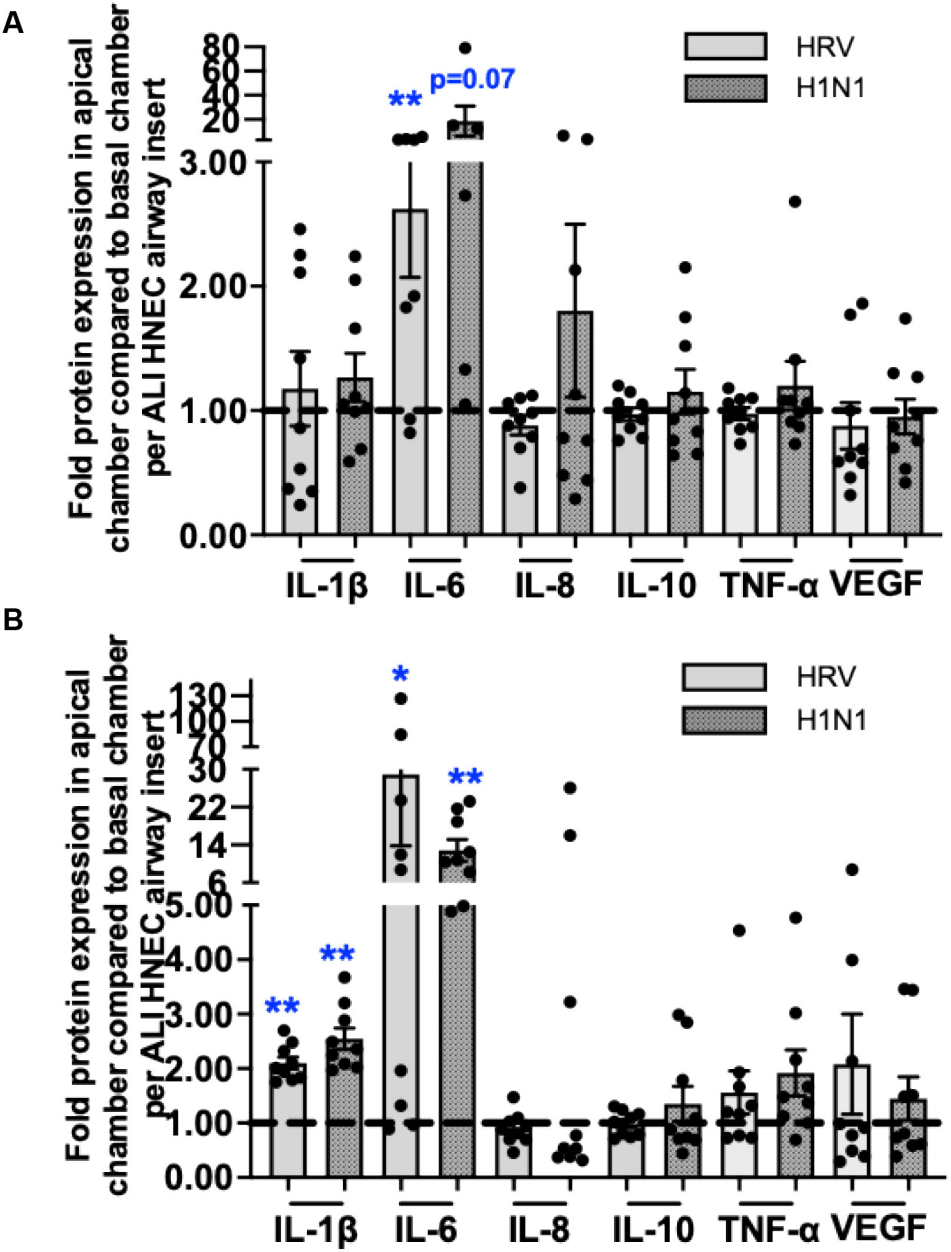
Impact of HRV and influenza infection on secretion of proinflammatory cytokines in apical versus basal surface in ex vivo model of nasal airways. Human nasal epithelial cells (HNECs) from three human donors were prepared and infected with HRV and H1N1 for 24 **(A)** and 48 **(B)** h, as described in the methods. The mean value of each protein measurement in each apical cell culture supernatant was normalized to the mean value of protein content in the same basal cell culture supernatant of the same ALI well. All panels show summary data as the mean ± standard error of the mean (SEM) from at least 3 biological replicates per donor. Three HNEC ALI cultures were prepared per human donor, and data from 3 donors are shown. Each data point represents the average of at least two technical replicates per one biological sample. Statistical comparison between the uninfected control group and each experimental group was performed using a two-tailed Mann–Whitney test and is indicated by a blue asterisk. A statistical comparison between two non-control experimental groups was performed using a two-tailed Mann–Whitney test, and the result is indicated by a black asterisk. The *p*-value for each comparison is shown above each column (*p < 0.05, **p < 0.01, ***p < 0.001). Alt Text: Graph and data showing measures of nasal airway inflammation in apical versus basal surfaces of human nasal airway cell culture models infected with influenza or human rhinovirus over 48 h compared to uninfected cell cultures.

To account for the complexity of host responses among different human donors and also to minimize experimental variability, the detected protein levels in the apical surface of each hNEC ALI insert were normalized by the respective protein levels in the basal surface of the same hNEC ALI insert, and data were expressed as a fold of apical to basal protein levels. We found that both HRV16 and H1N1-PR8 induced similar upregulation of apical and basal IL-8, IL-10, TNF-α, and VEGF at both 24 h.p.i (Figure 4A) and 48 h.p.i (Figure 4B). Notably, both HRV16 and H1N1-PR8 induced much higher upregulation of apical IL-6 secretion than basal secretion at both 24 h.p.i (Figure 4A) and 48 h.p.i (Figure 4B). Finally, both HRV16 and H1N1-PR8 induced much higher upregulation of apical IL-1β secretion than basal secretion at 48 h.p.i (Figure 4B) but not at 24 h.p.i (Figure 4A).

## 4 Discussion

Herein, using a reproducible ALI cell culture model of immortalized nasal airway cells where cells can be cryopreserved and utilized among independent research groups to minimize heterogeneity among different studies, we describe the differential *ex vivo* impact of independent respiratory viruses on established instigators of inflammation (IL-1β, IL-6, IL-8, IL-10, TNF-α) and angiogenesis and tissue remodeling (VEGF) during *ex vivo* infection of nasal airway cell cultures with two independent common respiratory viruses (HRV and Influenza A). Using a novel *ex vivo* mucosal model of immortalized hNEC established from three human donors and cultured with an ALI interface, and employing a multiplex Luminex immunoassay, we found that both HRV16 and H1N1-PR8 consistently and similarly increased levels of apical and basal TNF-α, and VEGF. HRV16 differentially increased secretion of IL-1β, IL-6, IL-8, and IL-10 levels in apical or basal surfaces versus H1N1-PR8. Both HRV16 and H1N1-PR8 induced significant upregulation of VEGF, a mediator of angiogenesis, thereby contributing to airway remodeling. Both HRV16 and H1N1-PR8 induced substantially greater upregulation of apical IL-6 secretion than basal secretion, suggesting that IL-6 plays a more prominent inflammatory role at the apical mucosal surface of the nasal airway epithelium. We demonstrate that this novel *ex vivo* culture system is a unique model that supports the controlled, standardized cultivation of HNECs under physiologically relevant polarization, enabling laboratory-based studies and further experimentation to explore the complexities of host-virus interactions in the nasal airway.

The immortalized nature of the NEC from multiple haplotypes of both genders eliminates the limitations associated with primary cell senescence and enables repeated culture studies, avoiding major limitations of other cell culture models, such as limited availability of human donors and substantial biological heterogeneity among donors. Immortalization of human epithelial cells with HPV16 E6/7 creates an intermediate between primary cell cultures and carcinoma-derived human cell cultures, enabling the use of easily replenished materials with a constant genetic background, through the enhanced lifespan of serially passaged heterogeneous cultures that preserve a greater capacity for cellular differentiation than neoplastic cell lines (Charles et al., 2019). Methodological outcomes for molecular analysis of RNA and proteins from both the apical and basolateral chambers model the nasal lumen and the host’s systemic compartments, respectively (Charles et al., 2019). While HPV E6/E7 immortalization extends the replicative lifespan of hNECs by bypassing senescence via inhibition of p53 and pRb, characterization in the literature demonstrates that these cells remain functionally equivalent to primary cells in their capacity for terminal differentiation in ALI cultures (Fulcher et al., 2005; Kelley et al., 2005; Kiyono et al., 1998; Liu et al., 2020; Vandermark et al., 2012; Viallet et al., 1994; Wazer et al., 1995; Zabner et al., 2003). Specifically, HPV-immortalized airway cells maintain the ability to form a pseudostratified epithelium with functional tight junctions, mucus production, and ciliary activity. Prior studies show that HPV-immortalized lines have conserved function and inflammatory signaling (Fulcher et al., 2005; Kelley et al., 2005; Kiyono et al., 1998; Liu et al., 2020; Vandermark et al., 2012; Viallet et al., 1994; Wazer et al., 1995; Zabner et al., 2003). Indeed, we confirmed that immortalization of hNEC did not affect their susceptibility to two independent respiratory viruses (HRV16 and H1N1), indicating that immortalization of hNEC did not compromise the ability to study host cellular responses in the context of respiratory viral infections.

Our findings confirm prior data showing that respiratory viruses induce nasal airway inflammatory cytokine production as early as 24 h post-infection. An important key finding is that IL-6 plays a more prominent inflammatory role on the apical mucosal surface of the nasal airway epithelium than on the basal surface. Our unique nasal airway cell culture model can dissect the differential polarized effects of respiratory viruses on nasal airway epithelium. Our data, in combination with prior literature, indicate that local IL-6 at relatively low doses can increase nasal secretions in patients with allergic rhinitis and mediate upper airway symptomatology (Gentile et al., 2001), suggest that the pro-inflammatory effect of respiratory viruses on nasal secretions may be mainly attributable to IL-6 upregulation rather than to other cytokines. However, we did not directly test downstream signaling effects *in vivo* to mechanistically link IL-6 with nasal symptomatology in viral respiratory infections.

Another novel finding is the previously understudied, major HRV-mediated upregulation of VEGF in the nasal airways. VEGF is identified as a principal proangiogenic factor that promotes the formation of new blood vessels from the existing vascular network, and its role in viral infections has mostly been studied in the context of oncogenic viruses (Alkharsah, 2018). Prior *in vitro* studies with epithelial cells have failed to demonstrate VEGF production following rhinovirus infection (De Silva et al., 2006; Lee et al., 2000). The pathogenesis of a “runny nose” is poorly understood and has been ascribed to the release of kinins (De Silva et al., 2006). The potent effects of the angiogenic factor VEGF on vascular permeability have been characterized and found to be more than 1,000-fold greater than histamine (Senger et al., 1990). Thus, the production of VEGF by independent respiratory viruses may explain some of the explosive vascular leakages found 24–72 h after initial exposure to respiratory viruses, since secretion by both apical and basolateral surfaces from HNECs may induce angiogenesis and tissue remodeling in deeper tissue layers and amplify the recruitment of pro-inflammatory cell populations (Lee et al., 2004). The use of our physiologically relevant mechanistic hNEC ALI model underscores the importance of studying proinflammatory responses in the context of a ciliated, polarized, multilayered airway epithelium and explains the limitations of prior negative findings on HRV and VEGF in studies that used only 2D *in vitro* cell culture models (De Silva et al., 2006; Lee et al., 2000).

Another novel finding is the understudied upregulation of VEGF by the major influenza virus in the nasal airways. Patients with influenza A/H1N1 infection and ARDS/AKI have an over-production of MCP-1, VEGF, and IP-10, possibly contributing to kidney injury (Bautista et al., 2013). Mechanistic studies have shown that influenza induces lung lymphangiogenesis *in vivo* during severe lung infection (Crossey et al., 2024). However, very limited data exist on whether influenza can directly upregulate VEGF production in the nasal airway epithelium. To our knowledge, our data are among the first to demonstrate that influenza A directly upregulates VEGF expression in human nasal airway epithelium, warranting further mechanistic investigation.

Our study has limitations. We did not study all possible proinflammatory mediators of tissue remodeling and the impact of other respiratory viruses on hNEC inflammation. Instead, we focused on key mediators of inflammation based on an established multiplex Luminex immunoassay. We did not perform a detailed study of time-dependent (infection time points) and dose-dependent (different virus inocula) effects of HRV and H1N1 on the secretion of proinflammatory airway mediators, nor did we mechanistically study the downstream signaling pathways of these mediators. Despite these limitations, our *ex vivo* studies provide novel insight into the pathogenesis of *early* nasal airway inflammation and tissue remodeling in respiratory viral infections.

In conclusion, our data showed that both HRV16 and H1N1-PR8 consistently and similarly increased the levels of apical and basal TNF-α and VEGF, while differentially impacted the levels of apical or basal IL-1β, IL-6, IL-8, and IL-10, during early infection in a novel polarized ex vivo culture system of primary human nasal epithelial cells. Both HRV16 and H1N1 induced significant upregulation of VEGF, which mediates angiogenesis and contributes to airway remodeling. Both induced preferential apical IL-6 secretion, indicating enhanced mucosal inflammatory signaling. This model is a unique model that enables controlled investigation of early nasal epithelial host–virus interactions in physiologically relevant polarization, allowing for lab-based causation studies and further experimentation to explore the complexities of host-virus nasal airway interactions.

## Data Availability

The original contributions presented in this study are included in the article/Supplementary material, further inquiries can be directed to the corresponding author.
