# Supplementary material for: Ex vivo modeling of nasal epithelial airway inflammation in respiratory viral infections
Source: Front Microbiol. 2026 Jun 3;17:1819581. doi: 10.3389/fmicb.2026.1819581 (PMC13272445; doi:10.3389/fmicb.2026.1819581)
Supplement: Supplementary file 1 [file Data_Sheet_1.PDF]

Supplemental material

Supplemental Figure 1

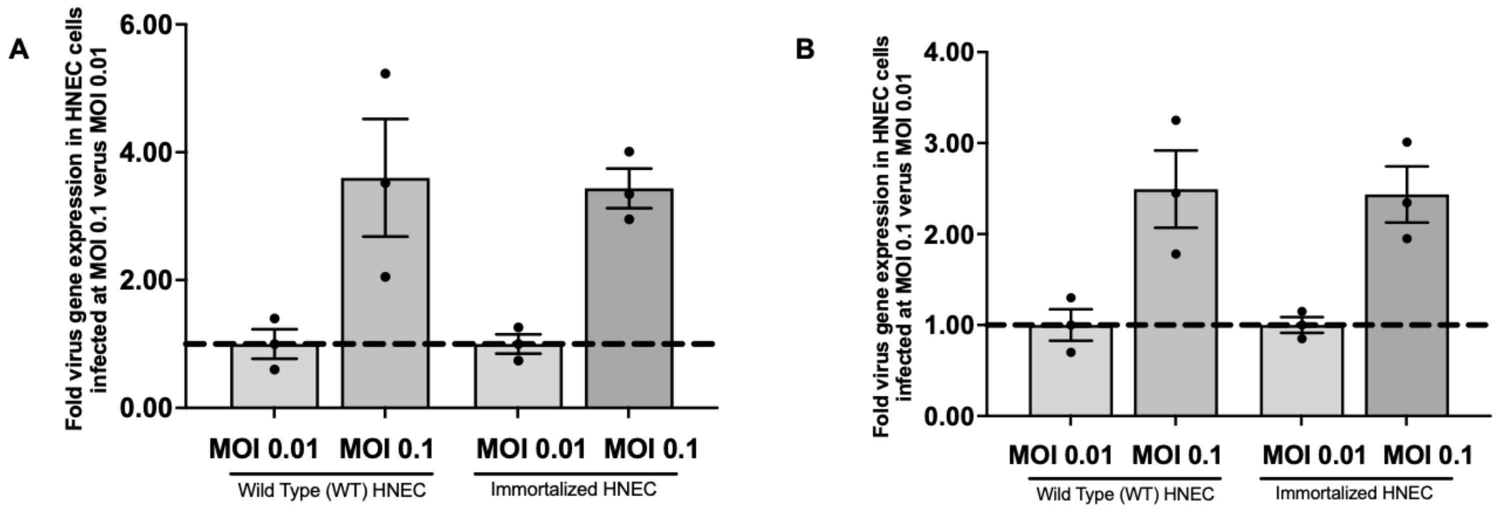

Supplemental Figure 1: Immortalization of human nasal epithelial cells (HNECs) does not affect their susceptibility to HRV and influenza infection in vitro. Immortalized human nasal epithelial cells (HNECs) from three human donors and wild-type (WT) primary HNECs from three human donors (commercially available) were prepared and infected with human rhinovirus (HRV16) (**A**) and H1N1 (**B**) for 24 hours, as described in the methods at multiplicity of infection (MOI) 0.01 and 0.1. Relative infectability (intracellular virus gene levels), over a ten-fold dose titration (MOI 0.1 versus 0.01), was determined by qPCR. Each data point represents the average of at least two technical replicates per one biological sample. Statistical comparisons between the experimental groups (MOI 0.1 in WT versus immortalized HNEC cells) were performed using a two-tailed Mann–Whitney test.
